# The Change of Electronic Transport Behaviors by P and B Doping in Nano-Crystalline Silicon Films with Very High Conductivities

**DOI:** 10.3390/nano6120233

**Published:** 2016-12-03

**Authors:** Dan Shan, Mingqing Qian, Yang Ji, Xiaofan Jiang, Jun Xu, Kunji Chen

**Affiliations:** 1 National Laboratory of Solid State Microstructures and School of Electronic Science and Engineering and Collaborative Innovation Center of Advanced Microstructures, Nanjing University, Nanjing 210093, China; shandnju@126.com (D.S.); hilarymoney@163.com (M.Q.); yangjimtz@163.com (Y.J.); jiangxiaofannju@163.com (X.J.); kjchen@nju.edu.cn (K.C.); 2 School of Electronic and Information Engineering, Yangzhou Polytechnic Institute, Yangzhou 225127, China

**Keywords:** carrier transport, doped, temperature-dependent Hall measurement

## Abstract

Nano-crystalline Si films with high conductivities are highly desired in order to develop the new generation of nano-devices. Here, we first demonstrate that the grain boundaries played an important role in the carrier transport process in un-doped nano-crystalline Si films as revealed by the temperature-dependent Hall measurements. The potential barrier height can be well estimated from the experimental results, which is in good agreement with the proposed model. Then, by introducing P and B doping, it is found that the scattering of grain boundaries can be significantly suppressed and the Hall mobility is monotonously decreased with the temperature both in P- and B-doped nano-crystalline Si films, which can be attributed to the trapping of P and B dopants in the grain boundary regions to reduce the barriers. Consequently, a room temperature conductivity as high as 1.58 × 10^3^ S/cm and 4 × 10^2^ S/cm is achieved for the P-doped and B-doped samples, respectively.

## 1. Introduction

Nano-crystalline Si (nc-Si) films have attracted much attention since they can be used in many kinds of devices such as Si-based light emitters, thin-film solar cells, as well as nonvolatile memories [1,2,3,4,5,6]. For example, highly efficient electroluminescence devices based on nc-Si have been fabricated and the external quantum efficiency reaches as high as 1.1% [3]. Recently, nc-Si/SiO_2_ or nc-Si/SiC films have been used to develop all-Si-based thin-film solar cells and the graded-sized structures are proposed to improve the power conversion efficiency [7,8,9]. In order to further improve the device performance, active doping of P and B in nc-Si is necessary to obtain n- or p-type nc-Si materials with high conductivities [10,11]. However, doping in nc-Si is quite different from their bulk counterpart due to the so-called “self-purification effect”. It was reported that the dopants may have difficulty entering the core of nc-Si, especially nc-Si with small sizes [12,13,14,15]. So far, the doping influences on the electrical and luminescence properties of nc-Si films have been studied but a comparable study on the carrier transport process in nc-Si materials before and after doping is still lacking [10,16,17,18]. Therefore, it is necessary to further understand the fundamental carrier transport behaviors in nc-Si films both in un-doped and doped samples.

In our previous works, the electronic properties in nc-Si films were investigated by the temperature-dependent conductivity measurements [18,19,20]. It was found that the carrier transport properties in nc-Si films were strongly influenced by the film structures and it looked like the thermally activated conduction dominated the carrier transport progress. It was also found that the room temperature conductivity was gradually enhanced by doping due to the activation of dopants accompanying the formation of nc-Si particles. In the present work, the microscopic mechanisms of the carrier transport process in un-doped, P- and B-doped nc-Si films are evaluated via the temperature-dependent Hall mobility measurements. It is confirmed that the grain boundary plays an important role in the carrier transport process in un-doped nc-Si film, which can be well described by the previously proposed model. After the doping of P or B, the significant increase in conductivity and the reduction of conductivity activation energy indicate the electrically active doping in nc-Si crystals. The temperature-dependent mobility in doped nc-Si films suggests that there is significant suppression of the grain boundaries’ scattering after doping.

## 2. Materials and Methods

Un-doped, P- and B-doped hydrogenated amorphous Si films with various doping concentrations were prepared by plasma enhanced chemical vapor deposition system using gas mixtures of pure silane (SiH_4_, Nanjing, China), hydrogen (H_2_, Nanjing, China), phosphine (PH_3_, 1% diluted in H_2_, Nanjing, China) and diborane (B_2_H_6_, 1% diluted in H_2_, Nanjing, China). The flow rate of SiH_4_ was kept at 5 sccm (standard cubic centimeter per minute). The phosphorus and boron concentrations were changed by adjusting the flow rate of PH_3_ (*F*_P_) and B_2_H_6_ (*F*_B_), which controlled at 0 sccm (un-doped), 0.5 sccm, 1 sccm and 5 sccm, respectively. During the growth process, the gas-chamber pressure, substrate temperature and radio frequency power were 10 mTorr, 250 °C and 30 W, respectively. All the samples thicknesses are about 200 nm. After deposition, the samples were first dehydrogenated at 450 °C for 1 h and then thermally annealed at the temperature of 1000 °C for 1 h in nitrogen ambient. Quartz plates and mono-crystalline Si wafers were used as substrates for various measurements.

Raman scattering spectra were detected by a Jobin Yvon Horiba HR800 spectrometer (HORIBA Jobin Yvon Co., Paris, France) operating with 1800 g/mm grating. The excitation light source is Ar^+^ laser with a wavelength of 514 nm. The high resolution transmission electron microscopy (TEM) images were observed by a TECNAIF20 FEI high resolution transmission electron microscopy (FEI Co., Hillsboro, TX, USA). The bonding configurations were characterized by Thermo ESCALAB 250 X-ray photoelectron spectroscopy (Thermo Co., Middlesex, MA, USA) (XPS) system and the composition signal of 5 nm under the surface was detected after Ar^+^ etching. Temperature-dependent Hall measurements were made by LakeShore 8400 (LakeShore Co., Lorain, OH, USA) using van der Pauw (VDP) geometry with coplanar configuration by vacuum evaporation Al electrodes on thin films. All the contacts yielded ohmic behavior in the entire measurement and the measurement temperatures are chosen from the range of 20–550 K.

## 3. Results and Discussion

### 3.1. Nanostructure

**Figure 1:**
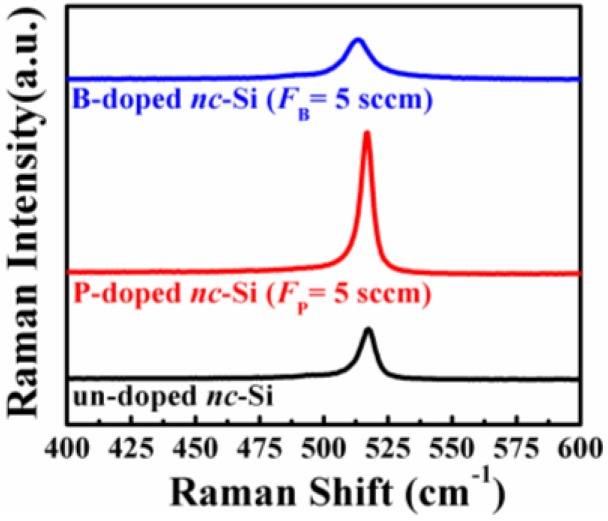
Raman spectra of un-doped nano-crystalline Si (nc-Si) film, P- and B-doped films with *F*_P_ = *F*_B_ = 5 sccm.

Raman spectroscopy was used to study the film microstructures of formed nc-Si films after annealing. As shown in Figure 1, a sharp and strong peak close to at 520 cm^−1^ appears for all the un-doped, P- and B-doped samples which indicates the crystallization of all the films. The crystalline fraction, *X*_C_, is determined based on Raman spectra by integrating the crystallized and amorphous Gaussian peaks [21]. It is found that the *X*_C_, which is about 85% for the un-doped sample, is increased above 90% after phosphorus doping. However, after boron doping, the *X*_C_ drops to about 78%. For the phosphorus doping, it was reported that the introduction of a great deal of phosphorus dopants into the Si host matrix could be helpful for the formation of four-coordination after annealing, which increases the structural ordering of the films and in turn promotes crystallization [22]. For the boron doping, it is suggested that an increase in the bond-angle and bond-length fluctuations as the boron dopant atoms are incorporated in the films causes the degradation of the short-range orderness [23], which may cause a slight reduction of crystallinity in B-doped nc-Si films.

**Figure 2:**
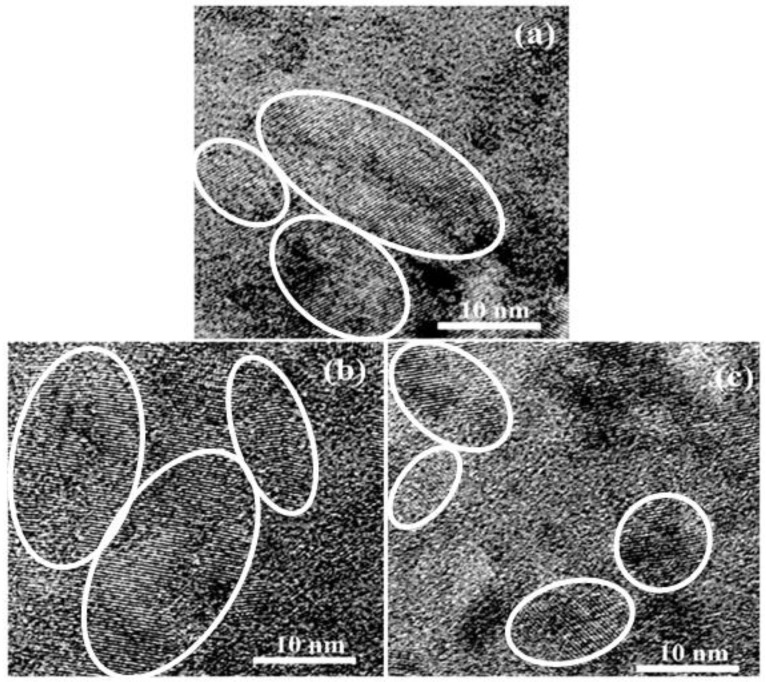
Transmission electron microscopy (TEM) images of (**a**) the un-doped nc-Si film; and (**b**) P-doped nc-Si film; and (**c**) B-doped nc-Si film.

In order to provide direct evidence for the presence of Si nanocrystals in the films, the formation of nc-Si was further demonstrated by cross-sectional TEM observations. As shown in Figure 2, the crystalline dots with various sizes and orientations can be clearly identified. From the TEM images in the various regions of the samples it can be found that the average grain size for all samples is about 15 nm. Compared with the un-doped nc-Si film, the P-doped sample presents a higher density of the Si nanocrystalline structure while the B-doped sample has a relatively low density.

**Figure 3:**
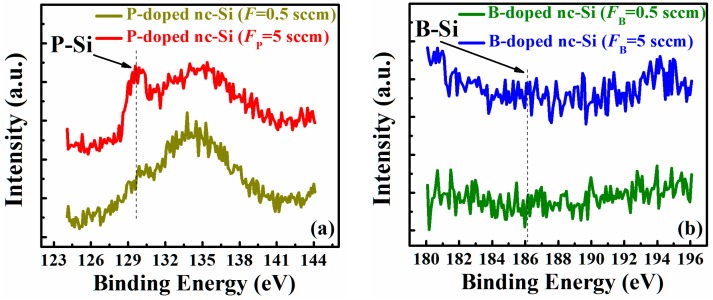
X-ray photoelectron spectroscopy (XPS) spectra of doped nc-Si films: (**a**) P-doped samples with different *F*_P_; and (**b**) B-doped samples with different *F*_B_.

The chemical bonding environments of P- and B-doped nc-Si films were investigated by XPS. Figure 3a,b shows the P 2p peak and the B 1s peak of P- and B-doped nc-Si films with different *F*_P_ and *F*_B_. For the P-doped samples, the peaks around 129 eV and 134 eV can be attributed to the P–Si/P–P bonds and the plasmon loss peak of silicon, respectively [19]. The intensity of the P–Si peak is much stronger and increases with the *F*_P_. The P atom content is estimated to be 0.67% and 1.69% for the P-doped samples with *F*_P_ = 0.5 and 5 sccm, respectively, which is similar to that reported by other groups [24,25,26]. For the B-doped nc-Si films, the featureless spectra as given in Figure 3b are shown. The B–Si signal (~186 eV) cannot be identified for B-doped samples, which indicates that the doping concentration of B in nc-Si films is lower than the detectable limit (~0.1%) of our system due to the low detection sensitivity of the B element in the XPS measurement [27].

### 3.2. Temperature-Dependent Conductivity

**Figure 4:**
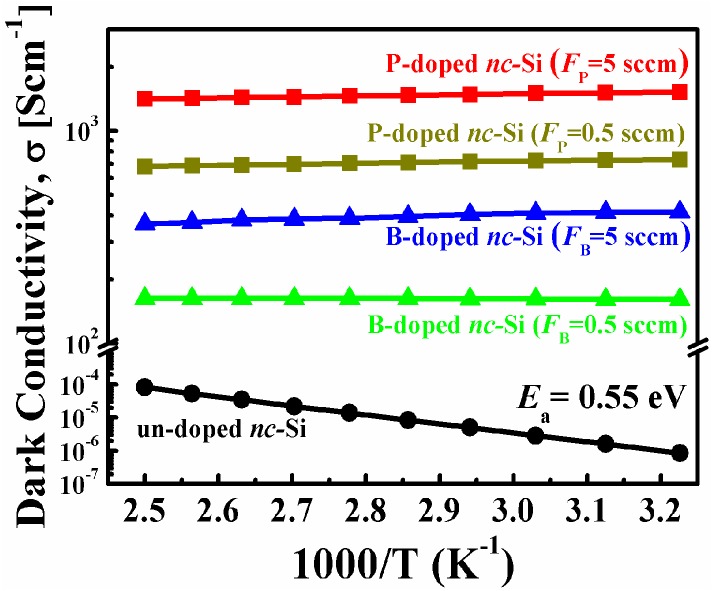
Temperature-dependent conductivities of nc-Si films with and without doping.

Figure 4 shows the temperature-dependent conductivity of un-doped, P- and B-doped nc-Si samples with the measurement temperature range of 310–400 K. The results can be well fitted by the Arrhenius plots σ = σ_0_*exp*(−*E*_a_/*k*_B_*T*), where σ_0_ is the conductivity prefactor, *k*_B_ is the Boltzmann’s constant and *E*_a_ is the conductivity activation energy. The conductivity activation energy *E*_a_ of the samples can be obtained using the slope of lnσ versus the 1/*T* curve. The un-doped sample exhibits activation energies around 0.55 eV, which is nearly half of the band gap of the crystalline silicon (1.12 eV). The measurement results indicate that the Fermi level is located at the mid-gap in the un-doped nc-Si sample. The linear behavior of σ versus 1000/*T* in the whole measurement temperature range for the un-doped sample suggests that the thermally activated transport of carriers dominates the carrier transport processes [20,28]. Meanwhile, both P- and B-doped samples show only very small activation energies close to 0 eV and the corresponding conductivity is almost temperature-independent. Similar results also reported previously that the conductivity activation energies were decreased significantly by P or B doping in nc-Si films [29]. It can be explained as the P or B dopants occupy the inner sites of nc-Si and the electrically active P or B dopants shift the Fermi level to the conduction (valence) band as in the bulk Si, and the room temperature conductivity is consequently clearly increased after doping. In our case, the dark conductivity is about 8.6 × 10^−7^ S/cm for un-doped nc-Si and it reaches as high as 1.58 × 10^3^ S/cm and 4 × 10^2^ S/cm for the P- and B-doped samples, respectively. Compared with any other works investigating the electronic properties of doped nc-Si films, our values of dark conductivities for P- and B-doped nc-Si films are significantly higher than those of previous reports [22,29,30]. Our experimental results also indicate that the nc-Si samples are easily heavily doped even at a relatively low gas doping ratio, i.e., *F*_B_ or *F*_P_ = 0.5 sccm. It can be understood that the doping concentration will be very high even if only a few dopants are incorporated into a one-Si dot with a diameter on the nanometer scale. It is also noted that the conductivities of B-doped nc-Si samples are lower than those of P-doped ones. It implies that the doping efficiency of B is lower than that of P in nc-Si materials, which can partly explain the absence of B-related XPS signals as shown in Figure 3b. It was reported that the phosphorus impurities tended to settle in the nc-Si core while the boron impurities preferentially occupied the surface sites of nc-Si dots [31]. S.H. Hong et al. also found that B atoms first substituted the inactive three-fold Si atoms in the grain boundaries of nc-Si and then substituted the four-fold Si atoms to realize electrically active doping [10]. Therefore, more P atoms than B atoms enter the inner sites of nc-Si and are ionized to contribute conduction carriers, which results in the low room temperature conductivity in B-doped nc-Si samples.

### 3.3. Temperature-Dependent Hall Mobility

In order to give a deeper insight into the carrier transport properties of the un-doped, P- and B-doped nc-Si films, temperature-dependent Hall mobilities were measured for all the samples. It is interesting to find that different behaviors of temperature-dependent Hall mobility are presented before and after doping. For the un-doped sample, the Hall mobility gradually increases as the temperature increases to 400 K and then decreases with further increasing the temperature. An increased Hall mobility with increasing temperature has been found in the polycrystalline silicon (poly-Si) films with a grain size on the micrometer scale. It was proposed that the grain boundaries of poly-Si played an important role in the carrier transport process and the carrier mobility was limited by the potential energy barriers at grain boundaries [32]. Such grain boundaries have a large number of defects which can trap the carriers and form the electrically charged states. The grain boundaries with electrically charged states impede the motion of carriers from one crystallite to another and the mobility is consequently reduced [33]. With increasing the temperature, the carriers gain the kinetic energy to overcome the potential barriers and the Hall mobility can be increased accordingly. The above discussion can be used to explain the increased mobility with a temperature up to 400 K in un-doped nc-Si film. Since the crystallinity of the un-doped nc-Si sample is as high as 85%, as revealed by Raman spectra, there are grain boundaries existing in the interface regions between the adjacent nc-Si dots. Thus, the scattering from the grain boundaries has an influence on the carrier mobility in un-doped nc-Si film.

**Figure 5:**
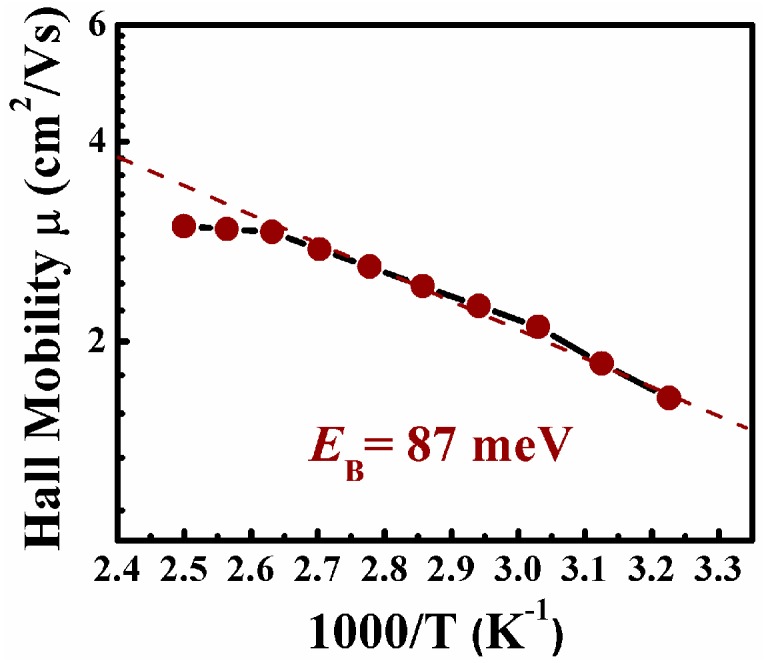
The Hall mobility as a function of the reciprocal temperature for the un-doped nc-Si film.

Seto et al. established the theoretical model to describe the grain boundaries’ scattering process and the mobility shows the thermally activated behavior as below:

(1) $$\mu_{H}\;(T)=\mu_{0}exp(-E_{B}/kT),$$

where $\mu_{0}$ is the exponential prefactor and *E_B_* is the activation energy which corresponds to the potential energy barrier height [34]. As shown in Figure 5, the relationship between the ln $\mu_{H}$ and 1000/*T* is given in the temperature range of 310–400 K. A good linear relationship indicated the experimental results were well fitted with Equation (1). The potential barrier height *E_B_* of the grain boundary can be deduced from the slope of the linear fitting which is about 87 meV.

**Figure 6:**
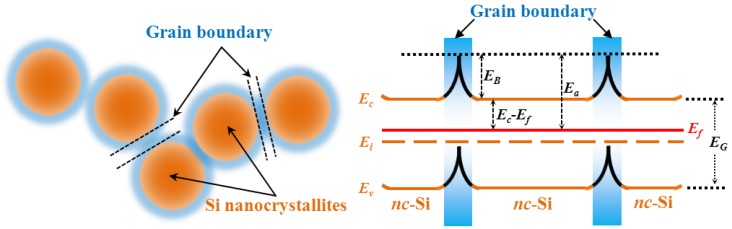
Schematic energy band diagram of the nc-Si films constituted by nano-crystalline phases and potential barrier caused by grain boundaries.

Considering the present nc-Si film with less amorphous components and potential energy barriers due to the grain boundaries between the crystalline components, the simplified energy band diagram is proposed as schematically plotted in Figure 6, where *E_C_* and *E_V_* represent the edge of the conduction band and valance band, while *E_f_* is the Fermi level, *E_B_* is the potential barrier height, and parts of the nc-Si regions near the surface become the depletion region, respectively. It is known that the carrier concentration $n_{0}$ and conductivity σ can be written as:

*n*_0_ ∝ *exp*(−(*E_C_* − *E_f_*)/*kT*), (2)

σ = *n*_0_*q*μ_H_ ∝ *exp*(−(*E_C_* − *E_f_*)/*kT*) × *exp*(−(*E_B_*)/*kT*) ∝ *exp*(−(*E_C_* − *E_f_* + *E_B_*)/*kT*), (3)

Therefore, the conductivity activation energy is *E*_a_ = *E_C_* − *E_f_* + *E_B_*, which represents the energy difference between the Fermi level and the top of the potential barrier as indicated in Figure 6. From the Hall measurements, the majority carrier (electron) concentration is about 1.7 × 10^12^ cm^−3^ for the un-doped nc-Si sample and the corresponding energy difference *E_C_* − *E_f_* = 0.44 eV. Consequently, *E*_a_ is about 0.53 eV by taking into account *E_B_* = 0.087 eV, which is in good agreement with the measured conductivity activation energy (~0.55 eV) as obtained before. Our experimental results suggest that the carrier transport in the temperature range of 310–400 K is mainly dominated by the potential barrier of the grain boundaries. When the temperature exceeds 400 K, the mobility is gradually decreased with the temperature as shown in Figure 5 since the carriers with sufficient kinetic energy can pass through the potential barrier at the high temperature. Therefore, the potential barrier is not a serious obstacle for the carriers and the phonon scattering governs the carrier transport process to limit the mobility of carriers, which results in the reduction of the Hall mobility with the temperature.

It is interesting to find that the behaviors of the temperature-dependent Hall mobility in doped nc-Si films are quite different from that of the un-doped one. The Hall mobility decreases monotonously with the temperature for both P- and B-doped samples in the whole measurement temperature range which indicates that the carrier transport process may not be dominated by the grain boundary scattering mechanism as in the un-doped sample. It may be ascribed to the reduction of the potential energy barrier height of the grain boundaries by doping, as suggested by the theoretical model to describe the transport process in doped micro-crystalline Si materials [33]. In our previous work, we studied the phosphorus dopants’ effect in nc-Si/SiO_2_ multilayers [35]. It was found that parts of the phosphorus dopants can passivate the surface states (dangling bonds) alongside the occupation of the inner sites of nc-Si dots. Therefore, the traps can be occupied by the added phosphorus (or boron) atoms and the corresponding mobility is enhanced after doping. The electron mobility for P-doped nc-Si film and the hole mobility for B-doped nc-Si film are about 30.3 cm^2^/V·s and 23.7 cm^2^/V·s at 310 K, respectively, which is obviously increased as compared with that of the un-doped nc-Si film (~1.6 cm^2^/V·s). Detailed studies are needed in the future to further understand the enhanced carrier mobility after doping.

**Figure 7:**
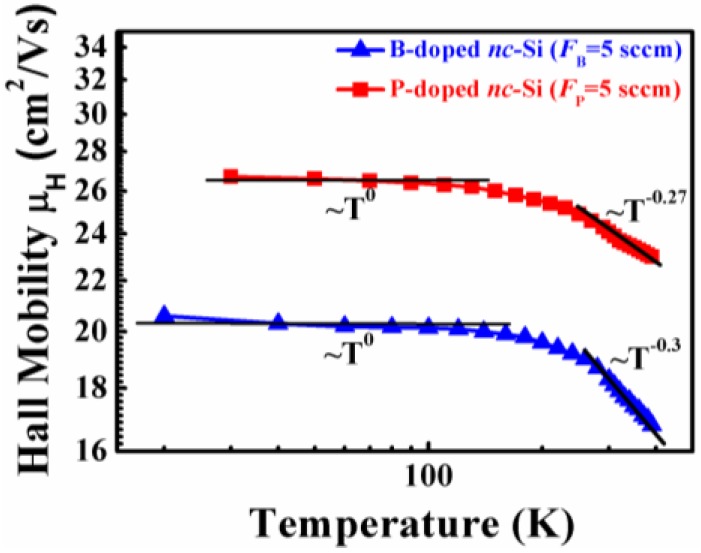
The Hall mobility, $\mu_{H}$, as a function of temperature for the P- and B-doped samples. The lines represent least-squares fits to $\mu_{H}$ (*T*) ∝ *T^n^*.

In order to further understand the carrier transport mechanism in doped nc-Si films, $\log\mu_{H}$ plotted as a function of logT in the given temperature range from 20 K to 400 K is shown in Figure 7. The temperature dependence of the Hall mobility can be described by the following equation:

μ_H_ (*T*) ∝ *T^n^*, (4)

Least-squares fits of the data yield *n* = 0 in the low-temperature region (20–100 K) and *n* = −0.3 in the high-temperature region (300–400 K) for the P- and B-doped samples with *F*_P_ and *F*_B_ = 5 sccm. In c-Si, typical scattering mechanisms are scattering at acoustic phonons, ionized and neutral impurities that yield values of −1.5, 1.5 and ≈0, respectively. The *n*-value of 0 for the P- and B-doped nc-Si films in the low-temperature region indicates that the carrier transport properties are controlled by the neutral impurities’ scattering mechanism which is derived from the un-ionized phosphorus or boron atoms. However, the exponents show the *n*-value of about −0.3 for the P- and B-doped nc-Si films in the high-temperature region. It is suggested that the carrier transport is dominated by a superposition of impurity scattering and acoustic phonon scattering within the high-temperature range.

## 4. Conclusions

The electronic transport behaviors of un-doped, P- and B-doped nc-Si films were prepared and their electronic properties were comparably studied. It is shown that the grain boundaries existing in nc-Si films influence the carrier transport process in un-doped samples. Based on the temperature-dependent Hall mobilities and conductivities, the height of the barrier induced by the grain boundary is estimated to be 87 meV. It is interesting to find that doping of P and B can reduce the grain boundary barriers which results in different behaviors of temperature-dependent mobilities of doped samples. Very high room temperature conductivity is achieved, which is 1.58 × 10^3^ S/cm and 4 × 10^2^ S/cm for P- and B-doped samples, respectively. Our present work revealed the microscope carrier transport behaviors of un-doped and P/B-doped nc-Si films and it is demonstrated that doping is still an effective way to improve the electronic properties of nc-Si films for device applications.
